# Growth hormone reduces retinal inflammation and preserves microglial morphology after optic nerve crush in male rats

**DOI:** 10.3389/fncel.2025.1636399

**Published:** 2025-09-05

**Authors:** Jerusa E. Balderas-Márquez, David Epardo, Lourdes Siqueiros-Márquez, Martha Carranza, Maricela Luna, José Luis Quintanar, Carlos Arámburo, Carlos G. Martínez-Moreno

**Affiliations:** 1 Departamento de Neurobiología Celular y Molecular, Instituto de Neurobiología, Campus Juriquilla, Universidad Nacional Autónoma de México, Querétaro, Mexico; 2 Departamento de Fisiología y Farmacología, Centro de Ciencias Básicas, Universidad Autónoma de Aguascalientes, Ags, Mexico

**Keywords:** growth hormone (GH), optic nerve crush (ONC), microglia, neuroinflammation, retina, proinflammatory cytokines, neuroprotection

## Abstract

**Introduction:**

This study investigates the neuroprotective role of growth hormone (GH) in modulating retinal inflammation and microglial responses following optic nerve crush (ONC) in male rats.

**Methods:**

Retinal inflammation and microglial activation were assessed at 24 h and 14 days post-ONC, with or without GH treatment (0.5 mg/kg, subcutaneously, every 12 h). Gene and protein expression of inflammatory markers (e.g., IL-6, TNFα, Iba1, CD86, CD206) were evaluated using qPCR, ELISA, and Western blotting. Microglial morphology was quantified using skeleton and fractal analysis of Iba1-stained retinal sections. Retinal structure and function were assessed via fundus imaging and optomotor reflex testing.

**Results:**

ONC induced significant increases in proinflammatory cytokines (IL-6, TNFα, IL-18) and microglial activation, characterized by reduced branching complexity and increased cell density. GH treatment significantly decreased proinflammatory cytokine levels, modulated microglial phenotype (CD86/CD206 expression), and preserved microglial morphology in the retina. Using the SIM-A9 microglial cell line, we further demonstrated that GH reduces NFκB pathway activation and suppresses LPS-induced proinflammatory cytokine production. At 14 days post-injury, GH-treated retinas exhibited reduced optic nerve size and improved optomotor responses, indicating both structural neuroprotection and functional recovery.

**Discussion:**

Overall, GH mitigates ONC-induced retinal inflammation by reducing proinflammatory signaling and preserving microglial architecture, thereby protecting retinal integrity and function. These findings highlight the potential of GH as a therapeutic agent for retinal neurodegenerative conditions.

## Introduction

Neuroinflammation refers to the inflammatory response to an injury within the nervous system, primarily involving the activation of glial cells, including astrocytes and microglia, and, in some cases, the infiltration of immune cells that contribute to the healing process. These glial cells play an important role in immune surveillance and mediate the inflammatory response by releasing cytokines, chemokines and other mediators (Garden and Möller, 2006; DiSabato et al., 2016; Kaur and Singh, 2023). In the retina, inflammation is initially a protective process aimed at defending against injury with mechanisms designed to contain and repair damage, promoting tissue repair; this process can be triggered by several factors such as trauma or microenvironmental changes (Quaranta et al., 2021; Ishikawa et al., 2023). If prolonged, retinal inflammation may lead to vision loss, driven by cell death and the release of proinflammatory cytokines [e.g., interleukin 1β (IL1β), interleukin 6 (IL6) and tumor necrosis factor alpha (TNFα)], which can induce oxidative stress, promote blood-retina barrier breakdown, and initiate apoptotic pathways in retinal cells. These inflammatory mediators not only exacerbate neuronal damage but also contribute to chronic retinal pathologies (Madeira et al., 2015; Silverman and Wong, 2018; Olivares-González et al., 2021; Fan et al., 2022; Kaur and Singh, 2023).

Microglia, the resident immune cells of the retina, play a significant role in retinal inflammation and are mainly located in the retinal ganglion cell layer (RGCL) and inner plexiform layer (IPL) (Au and Ma, 2022; Fan et al., 2022). Under normal conditions, microglial cells constantly monitor the retinal microenvironment and maintain homeostasis through interactions with other retinal cells (Silverman and Wong, 2018; Rashid et al., 2019; Fan et al., 2022). However, upon activation, microglial cells undergo morphological changes, which are often linked to apoptosis and neuronal degeneration. Depending on the stimulus, activated microglia can adopt either a classical (M1; proinflammatory) or alternative (M2; neurotrophic) phenotype. M1 microglia are associated with the release of proinflammatory cytokines, whereas M2 microglia promote the expression of anti-inflammatory cytokines (Kobayashi et al., 2013; Orihuela et al., 2016; Yusuying et al., 2022). Microglial activation and phenotypic changes involve alterations in their morphology, characterized by the retraction of branching process, which include junctions, end points, and overall complexity (Morrison and Filosa, 2013; Heindl et al., 2018; Ramírez et al., 2020; Godeanu et al., 2023). These changes are associated with their functional role in central nervous system (CNS) diseases and serve as indicator of microglial actions. For instance, M1 state tend to have shorter, less ramified processes, corresponding to their proinflammatory role, whereas M2 microglia exhibit more complex, and highly ramified morphologies, indicative of their tissue repair function (Ramírez et al., 2020; Lier et al., 2021; Fujikawa and Tsuda, 2023).

Growth hormone (GH) is a neurotrophic and neuroprotective factor in the CNS that functions throughout life, from embryonic development to maturity and senescence (Scheepens et al., 2001; Sanders et al., 2011; Waters and Brooks, 2012; Martinez-Moreno et al., 2018). During eye development, GH regulates the waves of developmental apoptosis critical for proper retinal formation, as shown in early chicken embryos where intravitreal interference with GH mRNA increased cell death (Sanders et al., 2011). Post-mortem human studies reveal that healthy retinal ganglion cells (RGCs) exhibit GH immunoreactivity, while apoptotic cells lack it, underscoring GH’s pro-survival role in the adult retina (Sanders et al., 2009). GH receptors (GHRs) are present in the retina across all vertebrate groups studied, activating the canonical JAK/STAT pathway alongside MAPK, PI3K/Akt, and Notch signaling pathways (Sanders et al., 2011; Fleming et al., 2019). Despite the ubiquitous distribution of GHRs in neuroretinal cells, the specific role of GH bioactivity in mature visual physiology remains poorly understood. However, both the receptor and ligand are present in the retina, optic nerve, tract, and tectum (Baudet et al., 2007). While GH is primarily produced by the pituitary gland, it is also synthesized in various extrapituitary tissues, like the CNS (Harvey, 2010; Arámburo et al., 2014) including the retina (Harvey et al., 2016).

The interplay of endocrine, paracrine, and autocrine GH actions in the visual system remains complex and deserves further investigation. Our group recently demonstrated that GH promotes synaptic restoration after optic nerve crush (ONC), resulting in functional recovery as evidenced by electroretinogram (ERG) studies (Epardo et al., 2024). Retinal function recovery correlates with axonal regeneration in the optic nerve, highlighting the therapeutic potential of GH in neuroregeneration (Epardo et al., 2024). To date, there is no direct evidence of GH’s effects on retinal glial cells; however, it is likely that its beneficial actions in the neural retina involve interactions with astrocytes, microglia, and Müller cells.

This study aims to investigate the effects of GH on microglia-mediated inflammation in the retina, with the goal of identifying new therapeutic strategies. Specifically, it examines the impact of GH action on cytokine expression and microglial activation, including phenotypic changes associated with neuroinflammation and optokinetic reflex recovery.

## Materials and methods

### Animals

We employed male Wistar rats (*Rattus novergicus domestica*) aged 6 weeks with an average weight of 250 gr across all experimental protocols. The animals were housed under standardized conditions, maintaining a 12-h light–dark cycle (12:12), and *ad libitum* access to water and standard rodent chow throughout the procedures. All experimental manipulations adhered rigorously to the animal handling guidelines outlined by the Association for Research in Vision and Ophthalmology (ARVO). Prior to implementation, each procedure was subjected to meticulous review and approval by the Institute of Neurobiology’s Research Ethics Committee at the National Autonomous University of Mexico, ensuring compliance with ethical standards and regulatory requirements (protocol 122A). Experimental interventions were exclusively directed on the left eye of each subject, while maintaining the right eye as an internal control.

### Optic nerve injury and experimental design

**Figure 1:**
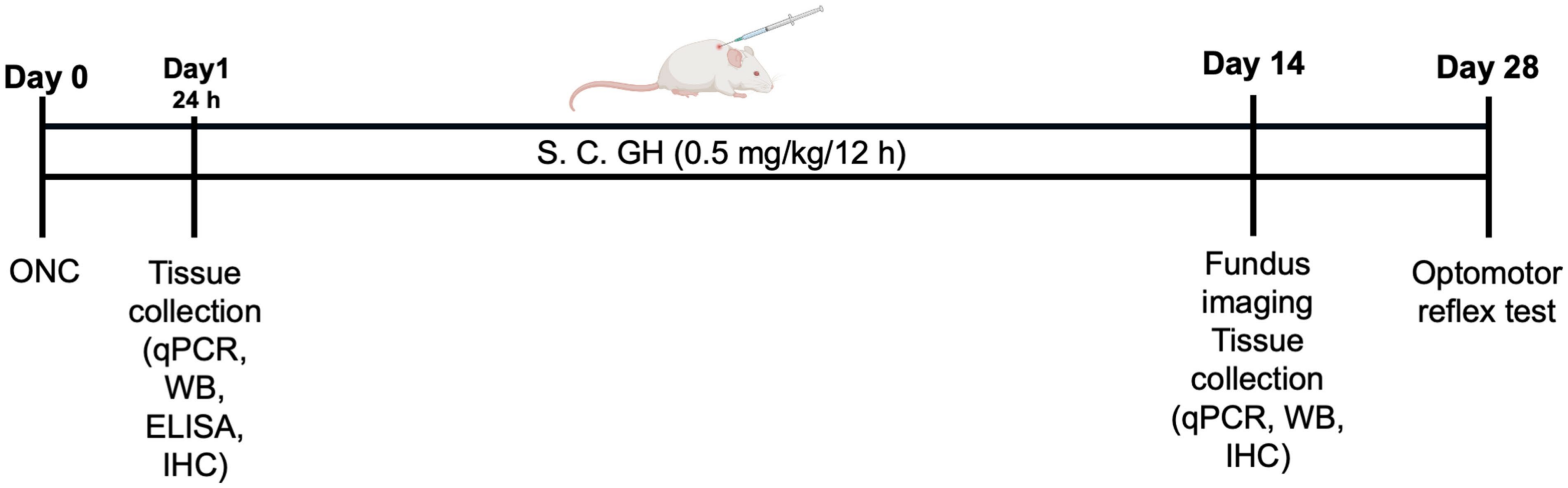
Schematic representation of the experimental design. At least two independent experiments were conducted for each time point (24 h, 14 days, and 28 days).

Three experimental groups were formed in which animals with ONC, or sham surgery were performed on the left eye. They were anesthetized intraperitoneally using ketamine (80 mg/kg) and xylazine (8 mg/kg). To induce the ONC, an incision was made in the lower conjunctiva of the left eye, allowing access to the optic nerve. The extraocular muscles were separated, and the optic nerve was exposed using fine scissors. The optic nerve was crushed 2 mm behind the optic disc for 10 s, using self-closing forceps (Dumont Tweezers #7; Dumoxel) (Allen et al., 2021), taking care to preserve the ophthalmic artery. In the sham group, the same procedure was replicated, excluding manipulations of the optic nerve. After surgery, animals received subcutaneous injections of bovine recombinant growth hormone (bGH) (Boostin-S, Intervet) every 12 h at dose of 0.5 mg/kg (Figure 1; Aberg et al., 2009). Tissue samples were collected either 24 h or 14 days after ONC, with at least two independent experiments conducted at each time point. Retinas were collected, frozen at −80°C, and later processed for qPCR or protein analysis by Western blot or ELISA. For histological assessments, both eyes and optic nerves were promptly fixed as described below and preserved for further analysis.

### Gene expression quantification by real time PCR

At the predetermined time points (24 h or 14 days post-ONC), retinas were individually collected, frozen on dry ice, and stored at −80°C for later processing. Total RNA extraction was performed using the Zymo Direct-zol purification kit in combination with TRIzol reagent (Zymo Research Corp., Irvine, CA, United States), strictly following the manufacturer’s guidelines. RNA was analyzed using a NanoDrop Lite Plus spectrophotometer (Thermo Fisher Scientific, Waltham, MA, United States), where it was quantified and its purity assessed (A260/A280). Only samples with a purity ratio within the range of 1.9 and 2.1 were used for downstream applications. For complementary DNA (cDNA) synthesis, 1 μg of RNA was reverse-transcribed using the high-capacity cDNA reverse transcription kit (Applied Biosystems, Waltham, MA, United States) strictly following the manufacturer’s instructions. The resulting cDNA was stored at −20°C until further use. Gene expression of selected targets (detailed in Tables 1, 2) was assessed through qPCR using the QuantStudio system (Applied Biosystems) and SYBR green (Maxima; Thermo Fisher Scientific, Waltham, MA, United States) in a reaction volume of 10 μL. No-template controls were also included to detect contamination and avoid false positives, and each sample was analyzed in technical replicate. The cycling conditions included: 95°C for 5 min, and then 40 cycles of 95°C for 15 s, 60°C for 15 s, and 72°C for 15 s. A dissociation curve was run at the end of each qPCR experiment to confirm primer specificity. Validation curves were generated for all oligonucleotides by constructing standard curves based on serial dilutions. The standard curve was plotted with the logarithm (base 10) of cDNA dilution factor on the x-axis and Ct values on the y-axis. Linear regression was used to derive the equation of the curve (y = mx + b), from which the slope (m) was obtained. The efficiency values (E) were obtained using the formula: E = ((10^−(1/slope))−1)*100. Amplification efficiencies were ranged between 95 and 115%. All primers were designed using Primer-BLAST (NCBI) to span an exon–exon junctions to prevent amplification of genomic DNA. Primer specificity was confirmed by melt curve analysis and agarose gel electrophoresis, which showed a single amplicon of the expected size without primer-dimers. Relative mRNA levels were quantified using the 2^(^−ΔΔ^CT) method and normalized against ribosomal protein S18 (RPS18) mRNA as reference gene (Livak and Schmittgen, 2001), which was selected as the most stable housekeeping gene in this model based on NormFinder analysis, with a stability value of 0.015.

**Table 1:** Rat (*Rattus norvegicus*) oligonucleotides.

Gene	Function	Sense	Sequence	Tm (°C)	Amplicon (nt)	Size (bp)	NCBI accession number
Il1b	IL1β	F	CACCTCTCAAGCAGAGCACAG	78.5	773–851	79	NM_031512.2
		R	GGGTTCCATGGTGAAGTCAAC				
Il4	IL4	F	TGTAGAGGTGTCAGCGGTCT	77.9	334–403	70	NM_201270.1
		R	TCAGTGTTGTGAGCGTGGAC				
Il6	IL6	F	TCCTACCCCAACTTCCAATGCTC	75.7	559–637	79	NM_012589.2
		R	TTGGATGGTCTTGGTCCTTAGCC				
I10	IL10	F	GGGAGAGAAGCTGAAGACCC	81.7	337–462	126	NM_012854.2
		R	ACACCTTTGTCTTGGAGCTTATTA				
Il18	IL18	F	CAAAAGAAACCCGCCTGTGT	79.9	664–736	73	NM_019165.2
		R	CAGTCTGGTCTGGGATTCGT				
Tnf	TNFα	F	ACCACGCTCTTCTGTCTACTG	80.2	277–445	169	NM_012675.3
		R	CTTGGTGGTTTGCTACGAC				
Tnfrsf1a	TNFR1	F	GTCTCCCCATCGTGCCTG	81.1	241–434	194	NM_013091.2
		R	GGTTCCTTTGTGGCACTTGG				
Tnfrsf1b	TNFR2	F	TGCAACAAGACTTCAGACACCGTG	79.8	271–353	83	NM_130426.4
		R	AGGCATGTATGCAGATGGTTCCAG				
Nos3	eNOS	F	TGGCAGCCCTAAGACCTATG	82.9	3,246–3,488	243	NM_021838.2
		R	AGTCCGAAAATGTCCTCGTG				
Nos2	iNOS	F	CCTTGTTCAGCTACGCCTTC	80.2	1,673–1851	179	NM_001429940.1
		R	GGTATGCCCGAGTTCTTTCA				
Ptgs2	Cox2	F	TGTATGCTACCATCTGGCTTCGG	78.5	1,001–1,094	94	NM_017232.4
		R	GTTTGGAACAGTCGCTCGTCATC				
Vegfa	VEGF	F	GCAATGATGAAGCCCTGGAG	81.04	1,222–1,299	78	NM_001287114.1
		R	GGTGAGGTTTGATCCGCATG				
Aif1	Iba1	F	GCCTCATCGTCATCTCCCCA	81.5	52–193	142	NM_017196.3
		R	AGGAAGTGCTTGTTGATCCCA				
Cd86.	CD86	F	TCAATAGCACTGCATACCTGCC	78.2	273–350	78	NM_020081.3
		R	GCCAAAATACTACGAGCTCACT				
Mrc1	CD206	F	ACTGCGTGGTGATGAAAGG	76.3	1,490–1,556	67	NM_001106123.2
		R	TAACCCAGTGGTTGCTCACA				
Tgfb1	TGFβ	F	TCGACATGGAGCTGGTGAAA	80.8	252–322	71	NM_021578.2
		R	GAGCCTTAGTTTGGACAGGATCTG				
Igf1	IGF1	F	ACCTTGCAAAAGTGGTCCTG	75.9	683–846	164	NM_178866.4
		R	AGGAATTTAGTGCAACCGAA				
Igf1r	IGF1R	F	GCCGTGCTGTGCCTGTCCTAAAAC	83.1	3,049–3,237	189	NM_001414181.1
		R	GCTACCGTGGTGTTCCTGCTTCG				
Rps18	RPS18	F	TTCAGCACATCCTGCGAGTA	76.3	71 – 206	136	NM_213557.1
		R	TTGGTGAGGTCAATGTCTGC				

**Table 2:** Mouse (*Mus musculus*) oligonucleotides.

Target gene	Function	Sense	Sequence	Tm (°C)	Amplicon (nt)	Size (bp)	NCBI accession number
Il1b	IL1β	F	GCAACTGTTCCTGAACTCAACT	74.7	91–179	89	NM_008361.4
		R	ATCTTTTGGGGTCCGTCAACT				
Il6	IL6	F	CCAGTTGCCTTCTTGGGACT	79.6	109–209	101	NM_031168.2
		R	GGTCTGTTGGGAGTGGTATCC				
Tnf	TNFα	F	CTCCAGGCGGTGCCTATGT	79.6	245–311	67	NM_013693.3
		R	GAAGAGCGTGGTGGCCC				
Tgfb1	TGFβ	F	CCGAAGCGGACTACTATGC	79.8	1,163–1,230	68	NM_011577.2
		R	ATAGATGGCGTTGTTGCGGT				
Rn18 s	18 s	F	ACCCGTTGAACCCCATTCGTGA	79.6	1,585–1743	159	X01117.1
		R	GCCTCACTAAACCATCCAATCGG				

### Enzyme-linked immunosorbent assay (ELISA)

Retinas were microdissected from enucleated eyes. Protein extraction from retinal tissue was performed by homogenization using a GE 130 PB sonicator (Cole-Parmer, Vernon Hills, IL, United States) at an amplitude setting of 10 for 30 s in RIPA buffer (Abcam, Cambridge, United Kingdom), supplemented with a complete protease inhibitor cocktail (Roche, Basel, Switzerland).Eighty microgram of the extracted total protein from samples were placed in each ELISA plate well. The concentration of proinflammatory cytokines IL1β, IL6 and TNFα were analyzed using specific commercial enzyme-linked immunosorbent assay (ELISA) kits (IL1β, Ref: BMS630, Lot: 337105–009; IL6, Ref: ERA31RB, Lot: 724090623; TNFα, Ref: ERA56RB Lot: 709090623, Invitrogen, Waltham, MA, United States), following the manufacturer’s instructions, and absorbance was measured at 405 nm using an ELISA plate reader (Bio-Rad, Hercules, CA, United States). Cytokines levels were quantified in picograms per milliliter (pg/mL) and were expressed as the relative change compared to the sham group.

### Western blot analysis

Total protein extraction from SIM-A9 cell was performed through homogenization using a GE 130 PB sonicator (Cole-Parmer, Vernon Hills, IL, United States) at amplitude of 10 for 30 s in RIPA buffer (Abcam, Cambridge, United Kingdom), with the addition of a complete protease inhibitor cocktail (Roche, Basel, Switzerland). Proteins (40 μg per sample) were separated using 12% SDS-PAGE gels under reducing conditions, followed by transfer onto nitrocellulose membranes (Bio-Rad). To block non-specific binding, membranes were incubated with 5% non-fat milk (Bio-Rad) in TBS for 1 h at room temperature (RT, RT = 21°C). The membranes were then incubated overnight at RT with NF-kB antibody (Table 3) in TTBS (TBS with 0.05% Tween) containing 1% non-fat milk. After three washes in TTBS (3 × 5 min), the membranes were incubated for 2 h with HRP-conjugated secondary antibody (Table 3). Visualization of protein bands was achieved using the ECL blotting detection reagent (Amersham-Pharmacia, Buckinghamshire, United Kingdom), followed by exposure to autoradiography films (Fujifilm, Tokyo, Japan). Kaleidoscope molecular weight markers (Bio-Rad) were used to determine the approximate molecular weights of the proteins. Images were captured using a Gel Doc EZ Imager (Bio-Rad), and the immunoreactive band intensities were analyzed with Image Lab software (Bio-Rad). All target protein levels of immunoreactivity were normalized against *β*-actin as a loading control.

**Table 3:** Antibodies.

Target	Host/type	Dilution	Source	Cat. no.
Iba1	Goat/polyclonal	1:1,000	Abcam	AB5076
NFkB P65 (Phospo S536)	Rabbit/polyclonal	1:3,000	Abcam	AB86299
β -Actin	Mouse/monoclonal	1:5,000	Santa Cruz	SC-47778
MouseIgG	Rabbit/Alexa fluor 488	1:1,000	Invitrogen	A11029
Mouse IgG	Goat/Alexa fluor 594	1:1,000	Invitrogen	A11032
Mouse IgG	Goat/HRP conjugated	1:5,000	Invitrogen	A16160
Rabbit IgG	Goat/HRP conjugated	1:5,000	Invitrogen	65–6120

### Immunohistochemistry

The eyes were fixed in a 4% paraformaldehyde solution containing 3% sucrose diluted in PBS at 4°C, for 6 h. To optimize fixation, approximately 30 μL of this solution was injected into the eyes through the cornea using a 31-gauge insulin syringe. After fixation, the tissues underwent cryoprotection in PBS with progressively increasing sucrose concentrations of 10, 20, and 30% with 12 h of incubation in each solution. Subsequently, the tissues were frozen and mounted onto aluminum sectioning blocks using Tissue-Tek O. C. T (Sakura Finetek, Torrance, CA, United States). Retinal sections of 15 μm thickness were prepared using a cryostat (Leica CM3050 S, Buffalo Grove, IL, United States) and placed on glass slides. The eyes were sectioned along the naso-temporal axis at the equator. For immunohistochemical (IHC) analysis, the sections were first blocked with 5% Blotting-Grade Blocker non-fat dry milk (Bio-Rad) in PBS. They were then incubated overnight at 4°C with primary antibodies (as specified in Table 3), diluted in PBS containing 0.05% Triton X-100 and 1% non-fat dry milk. Following primary antibody incubations, sections were incubated for 2 h at RT with secondary fluorescent antibodies (Table 3) at a dilution of 1:1000 in the same solution, along with DAPI (100 ng/mL) (Sigma-Aldrich, St. Louis, MO, United States) to counterstain cell nuclei. Negative controls, in which the primary antibodies were omitted, were included for validation purposes. Image capture was performed using an Olympus BX51 fluorescence microscope (Olympus, Tokyo, Japan), and subsequent data analysis was completed using Image Pro 10 software (Media Cybernetics, Rockville, MD, United States).

### Morphological analysis of microglia

The steps for morphological analysis in this study were based on previous research that employed similar techniques to indicate microglial morphological changes (Young and Morrison, 2018; Green et al., 2022). Specifically, Iba1 staining was analyzed using the skeletal analysis and FracLac plugins in ImageJ (free ImageJ Fiji software, NIH, Bethesda, MD).

The Iba1-stained images were converted to black-and-white images, where 100 individual microglial cells were isolated for subsequent analysis. Each image underwent brightness and contrast adjustment to optimize the visualization of microglial branches. The threshold was then adjusted, and the image was converted to binary format, applying the close and remove outliers’ functions to refine the analysis. For single-cell skeletal analysis, the binary images were skeletonized and analyzed using the skeletal analysis plugin in ImageJ. This tool was employed to calculate the number of branches, junctions and end points in each microglia. For fractal analysis, the binary images of the microglial cells were converted to outlines. The FracLac plugin was used to perform box counting analysis with the grid design parameter “Num G” set to four. Several parameters were measured, including the fractal dimension (measure of the complexity of the pattern), lacunarity (measure of how the pattern fills space), density (number of pixels per area), span ratio (the longest length divided by the longest width), circularity (indicator of how circular the cell is), as well as the area and perimeter of each microglial cell.

### Fundus imaging

Fundus imaging was conducted 1 day after the completion of GH treatment. Pupils were dilated using a solution of tropicamide/phenylephrine (8 mg/50 mg/mL), and the corneas were kept hydrated with 5% dexpanthenol. Rats were anesthetized via intraperitoneal injection of ketamine (80 mg/kg) and xylazine (8 mg/kg). Brightfield images of the fundus were captured using a Micron IV fundus camera (Phoenix Research Laboratories, Bend, OR, United States). Following image acquisition, optic nerve diameters were measured using ImageJ software, and the relative diameter was calculated in comparison to the sham group.

### Optomotor reflex test

The optomotor reflex test was conducted 2 weeks after the completion of GH treatment. This interval was necessary because the animals did not reliably perform the task immediately after treatment, likely due to the handling associated with the experimental procedures. The visual function of conscious, unrestrained rodents was evaluated using a virtual optomotor task, specifically assessing the optomotor kinetic tracking (OKT) response to a visual stimulus. This was performed using the OptoMotry system (Cerebral Mechanics, Inc., Lethbridge, AB, Canada) (Redfern et al., 2011; Bretz et al., 2020).

For the test, rats were placed on an elevated platform inside the chamber. A video camera positioned above the animal provided real-time images, which were monitored by a trained observer on a desktop computer screen. The OptoMotry system projected a virtual rotating cylinder displaying a pattern of white and black lines. The rats were allowed to move freely on the platform. When a grating pattern, perceptible by the rat, was projected, the test commenced. A trained observer assessed whether the rat tracked the pattern by moving its head in the direction of the visual stimuli. The grating frequency (cycles per degree; c/d) was adjusted based on the observer input, if the rat was observed to track the pattern, the frequency was increased, and if not, it was decreased. The software automatically determined the endpoint of the test when changes between successive readings became minimal. The contrast of the stimuli was set at 100%, and the rotation speed at 12 degrees/s. If a rat slipped or jumped off the platform during the test, it was gently returned to the platform and the test resumed. The rats were generally tested during the early hours of their daylight cycle. The primary observer was masked to the treatment conditions, and a second trained observer independently confirmed the tracking assessments to ensure consistency. The optomotor response in rodents is known to be asymmetric. Therefore, the visual capabilities of each eye were assessed separately by rotating the stimulus in different directions: clockwise rotation tested the left eye (with ONC), while counterclockwise rotation tested the right eye (control) (Thomas et al., 2004).

### Anti-inflammatory effect of GH upon SIM-A9 cell line culture

The SIM-A9 microglial cell line (ATCC^®^ CRL-3265™, Manassas, VA, United States) was derived from a primary glial culture obtained from postnatal murine cerebral cortices. These cells underwent spontaneous immortalization, enabling continuous proliferation without requiring genetic or pharmacological interventions (Nagamoto-Combs et al., 2014; Dave et al., 2020). SIM-A9 cultures were maintained in Petri dishes using Dulbecco’s modified Eagle medium (DMEM) (Gibco, Life Technologies, Carlsbad, CA, United States) supplemented with 10% fetal bovine serum (FBS; Gibco, Thermo Fisher Scientific, United States) and antibiotics 10,000 units/mL of penicillin and streptomycin, and 25 μg/mL antifungal Fungizone (Gibco, Life Technologies). Cells were incubated at 37°C in a humidified 5% CO₂ atmosphere. Before initiating experiments, an equal number of cells were seeded in multi-well plates, incubated overnight, and subjected to serum deprivation for 2 h prior to treatment.

For experimental treatments, SIM-A9 cells were seeded at a density of $3x10^{5}$ cells/well in 6-well culture plates. Cells were either untreated (control; ctrl) or treated with lipopolysaccharide (LPS; 10 ng/mL; from *Salmonella enterica* serotype Minnesota from Sigma-Aldrich) alone or in combination with GH (LPS + GH; 10 ng/mL and 100 nM, respectively; bGH, NHPP AF10325C, Harbor-UCLA Medical Center, Torrance, CA, United States). Treatments were performed for 30 min for WB analysis and 6 h for qPCR.

### Statistical analysis

Gene expression (qPCR), protein levels (ELISA and Western blot), optic cup diameter, optomotor tests, and microglial morphology were analyzed across two independent experiments. These assessments involved 6–10 animals for qPCR, 6–8 animals for protein levels, 8–11 animals for optic cup measurements, 5–6 animals for optomotor tests, and 100 microglial cells from 6 animals for morphological analysis. All data are presented as mean ± SEM. Normality of data distribution was assessed using the Shapiro–Wilk test, and homogeneity of variances was evaluated with the Brown-Forsythe test. Outliers were identified using the ROUT method with Q = 1%. For data that did not meet the assumption of equal variances, Welch’s ANOVA was applied. For data where equal variances were observed, one-way ANOVA followed by Fisher’s LSD post-hoc test was used to determine statistical significance between groups or treatments. All statistical analyses were performed using Prism 10 software (GraphPad, San Diego, CA, United States). Differences were considered statistically significant when *p*-values were less than 0.05. Statistical significance is indicated as follows: *p < 0.05; **p < 0.01; ***p < 0.001; ****p < 0.0001.

## Results

### GH induces antiinflammatory effects in the rat retina after ONC

**Figure 2:**
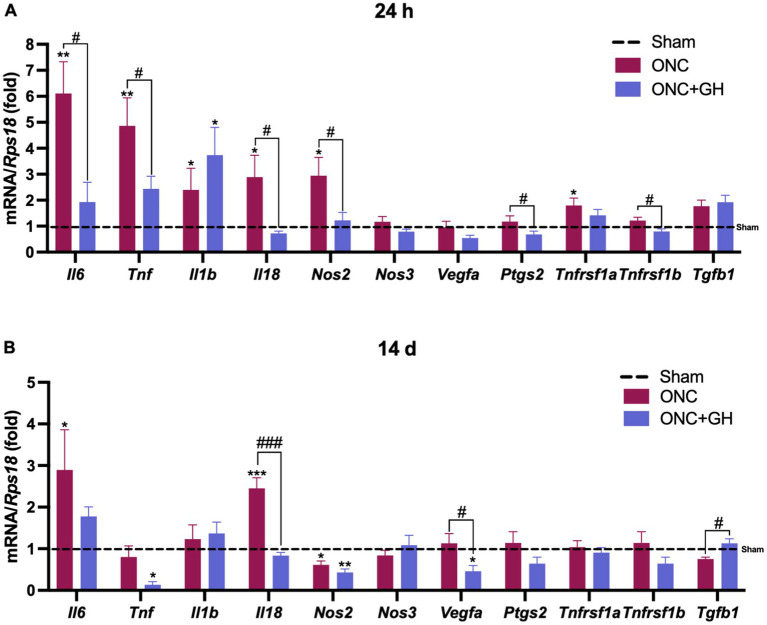
Cytokine mRNA expression in the rat retina at 24 h and 14 days post- ONC and GH treatment by qPCR. **(A)** Relative changes of proinflammatory cytokines (*Il6, Tnf, Il1b, Il18, Nos2, Nos3*), *vegfa*, *Ptgs2*, TNFα receptors (*Tnfrsf1a*, *Tnfrsf1b*), anti-inflammatory cytokine (*Tgfb1*) in the retina at 24 h post-ONC and GH treatment. **(B)** Relative changes of proinflammatory cytokines (*Il6, Tnf, Il1b, Il18, Nos2, Nos3*), *vegfa*, *Ptgs2*, TNFα receptors (*Tnfrsf1a*, *Tnfrsf1b*), anti-inflammatory cytokines (*Tgfb1*) in the retina at 14 days post ONC and GH treatment. Experimental groups: sham (control), optic nerve crush (ONC), ONC + GH. RPS18 was used as the reference gene. Results are shown as mean ± SEM (*n* = 7–9); asterisks show significant differences compared to the control (sham), while number signs indicate multiple comparisons among groups. ^*,#^*p* < 0.05; ^**^p < 0.01; ***, ^###^*p* < 0.001, determined by one-way ANOVA followed by Fisher’s LSD as *post-hoc* test.

The antiinflammatory effects of GH administration were evaluated by the expression levels of cytokines in the retina at 24 h and 14 days post-ONC. At 24 h (Figure 2A), ONC significantly increased *Il6* and *Tnf* expression (*p* < 0.01) compared to the sham group (dashed line). However, GH treatment significantly reduced *Il6* and *Tnf* expression (*p* < 0.05) compared to the ONC group. Both, ONC and ONC + GH groups significantly increased *Il1b* expression (*p* < 0.05). Similarly, *Il18* and *Nos2* expression were significantly upregulated (*p* < 0.05) by ONC compared to the sham group, and GH treatment significantly reduced (*p* < 0.05) the expression of these proinflammatory cytokines. The ONC group showed no significant effects on *Nos3* or *Vegfa* expression at this time point. Although no significant changes were observed in *Ptgs2* expression compared to the sham group, GH treatment resulted in a significant decrease (*p* < 0.05) compared to the ONC group. The expression of TNFα receptors was analyzed and *Tnfrsf1a* (proinflammatory) showed a significant increase (*p* < 0.05) in the ONC group compared to sham group, while *Tnfrsf1b* (pro-survival) did not show significant differences with the sham group but significantly decreased (*p* < 0.05) in the ONC + GH group compared to the ONC group. Neither treatment showed significant differences in *Tgfb1* expression at his time point.

Similarly, the expression of these cytokines was analyzed 14 days after ONC injury (Figure 2B). ONC injury significantly increased (*p* < 0.05) *Il6* expression compared to the sham group, while GH treatment reduced that effect and did not show any significant changes compared to sham group. *Tnf* levels in the ONC group were not different from sham, but GH treatment showed a significant decrease (*p* < 0.05) compared to the sham group. *Il1b* expression did not show significant changes in either the ONC or ONC + GH groups as compared to the control. In turn, *Il18* expression was significantly increased (*p* < 0.001) in the ONC group compared to sham, whereas GH treatment significantly reduced (*p* < 0.001) its expression compared to the ONC group. In contrast, *Nos2* expression was significantly decreased in the ONC group (*p* < 0.05) in comparison to sham, and GH treatment caused a further decrease (*p* < 0.01) compared to sham and was also different than ONC (*p* < 0.05), while *Nos3* expression showed no significant changes in either group. GH treatment significantly decreased *Vegfa* levels compared to both the ONC and sham groups, whereas the ONC group was no different than sham. *Ptgs2* and TNFα receptors (*Tnfrsf1a* and *Tnfrsf1b*) did not show significant changes between groups. Lastly, *Tgfb1* expression showed a significant increase (*p* < 0.05) in the GH-treated group compared to the ONC group.

### GH treatment regulates microglial activity after ONC at 24 h and 14 days

**Figure 3:**
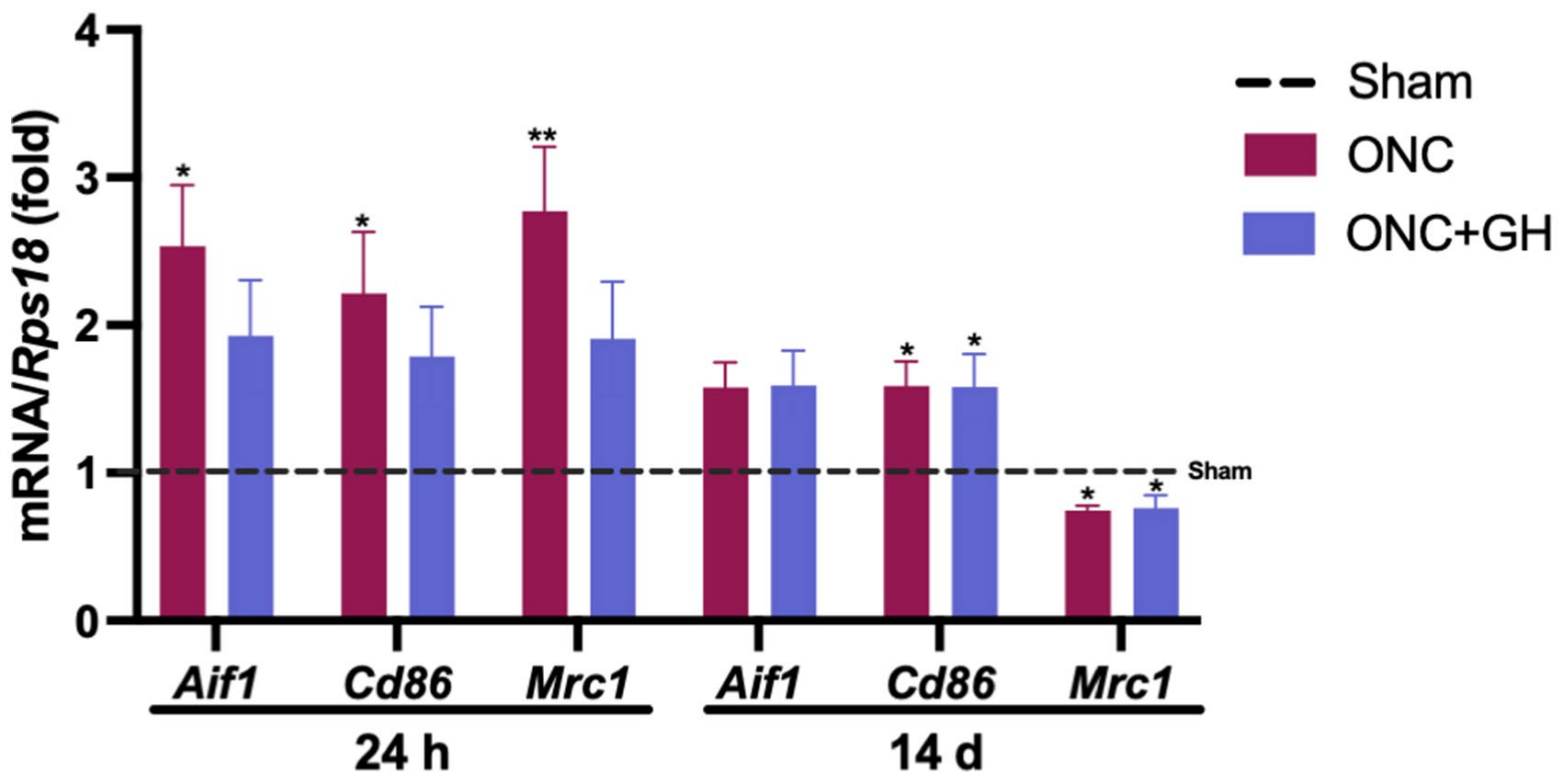
Microglial markers mRNA expression in the rat retina at 24 h and 14 days post-ONC and GH treatment by qPCR. Relative changes of microglial markers (*Aif1*, *Cd86*, and *Mrc1*) in the retina at 24 h and 14 days post-ONC and GH treatment. Experimental groups: sham (control), optic nerve crush (ONC), ONC + GH. *Rps18* was used as the reference gene. Results are presented as mean ± SEM (*n* = 7–9); asterisks indicate significant differences compared to the control (sham). ^*^p < 0.05; **p < 0.01, determined by one-way ANOVA followed by Fisher’s LSD as *post-hoc* test.

The damage caused by ONC in the retina affected cytokine expression, indicating an association with glial cell activity. Thus, microglial markers like Iba1 (*Aif1*), CD86 (*Cd86;* M1 phenotype), and CD206 (*Mrc1*; M2 phenotype) were analyzed at 24 h and 14 days post-ONC (Figure 3) by qPCR. At 24 h, ONC significantly increased *Aif1* and *Cd86* expression (*p* < 0.05), as well as *Mrc1* (*p* < 0.01) as compared to the sham group, whereas GH treatment did not show any significant differences compared to the control group.

On the other hand, after 14 days, no significant changes were observed in *Aif1* expression in any of the groups compared to control. However, both ONC and ONC + GH groups showed a significant increase (*p* < 0.05) in *Cd86* expression compared to sham. In contrast, *Mrc1* levels showed a significant decrease (*p* < 0.05) in both groups as compared to the control.

### Effect of GH upon cytokine proteins and microglial markers at 24 h post-injury

**Figure 4:**
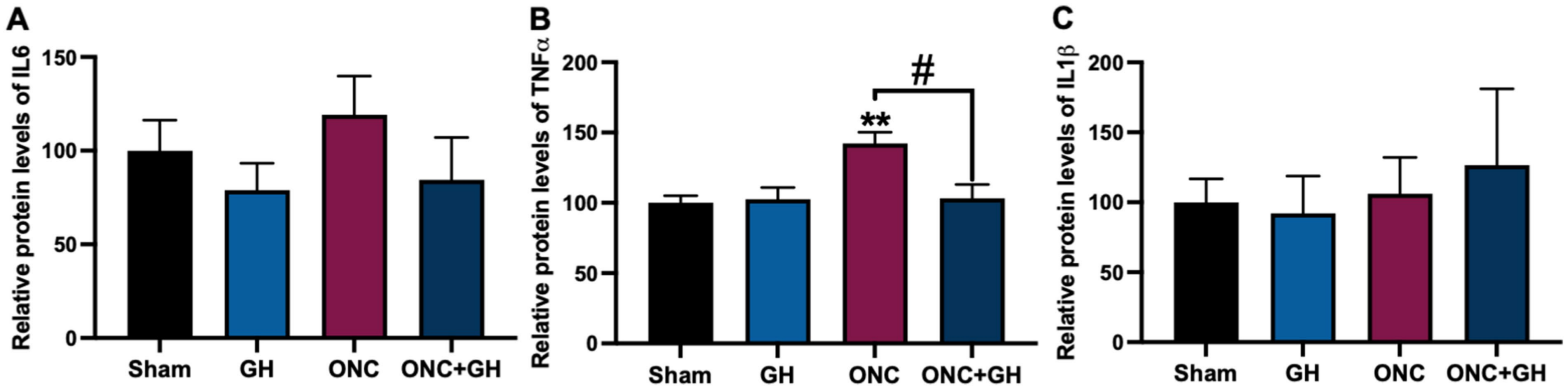
Protein levels of proinflammatory cytokines at 24 h post-ONC and GH treatment, as determined by ELISA. **(A–C)** Relative protein levels of IL6, TNFα and IL1β, respectively. Experimental groups: sham (control), growth hormone (GH), optic nerve crush (ONC), ONC + GH. Results are shown as mean ± SEM (*n* = 8); asterisks indicate significant differences compared to control (sham) and number signs indicate multiple comparisons among groups. ^#^*p* < 0.05; ^**^p < 0.01, determined by one-way ANOVA followed by Fisher’s LSD as *post-hoc* test.

Proinflammatory cytokine levels (IL6, TNFα and IL1β) in the retinas were measured 24 h post-ONC using ELISA (Figures 4A–C). No significant differences in IL6 levels were observed between the experimental groups (Figure 4A), although data showed a tendency to increase with ONC and to decrease with GH treatment. Conversely, TNFα levels were significantly elevated (*p* < 0.01) in the ONC group compared to the sham group, whereas GH treatment after ONC resulted in a significant decrease (*p* < 0.05) compared to the ONC group (Figure 4B). In turn, IL1β levels showed no significant differences among groups (Figure 4C).

### Microglial morphological changes in the retina 24 h after ONC and GH treatment

**Figure 5:**
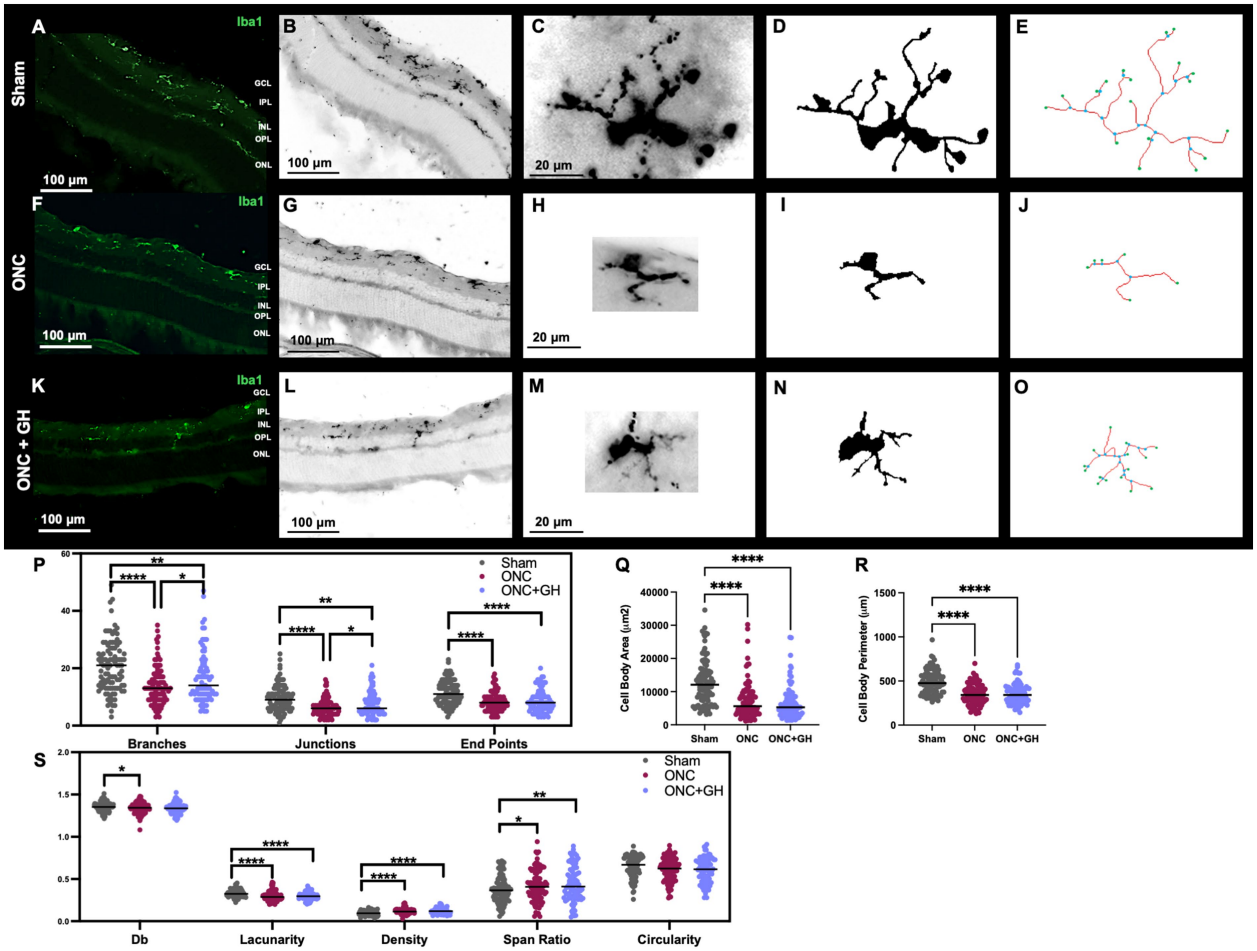
Microglial morphological changes in the rat retina 24 h after ONC and GH treatment, by using Iba1 immunohistochemistry. **(A,B,F,G,K,L)** Iba1 immunoreactivity in the retina; **(C,H,M)** isolated individual microglial cells; **(D,I,N)** binary images of the isolated individual microglial cells; **(E,J,O)** skeletonized images of individual microglia, where branches are in red, junctions in blue and endpoints in green; **(P)** quantification of skeleton analysis, including number of branches, junctions and end points; **(Q)** microglial cell body area; **(R)** microglial cell body perimeter; **(S)** fractal analysis showing fractal dimension (Db), lacunarity, density, span ratio, and circularity of microglia. Studies were performed using skeletal analysis and FracLac plugins in ImageJ. Experimental groups: sham (control), optic nerve crush (ONC), ONC + GH. Results are presented as individual data points with the mean indicated, and each group represented by a different color (*n* = 100 microglia, 6 animals). Asterisks indicate significant differences among groups. ^*^p < 0.05; ^**^p < 0.01; ^****^p < 0.001, determined by one-way ANOVA followed by Fisher’s LSD as *post-hoc* test.

To evaluate the effects induced by the treatments on microglia, IHC analyses were performed 24 h post-ONC (Figure 5) using a specific antibody against Iba1. Results revealed that, in the sham group, a clear Iba1-immunoreactivity (IR) was observed in the IPL, although some Iba1-IR was also present in the outer plexiform layer (OPL). However, following ONC, an important reduction in Iba1-IR was noticed in the OPL. Morphological changes were quantified using Iba1 IHC analysis (Figures 5A,B,F,G,K,L), where individual microglial cells were isolated from the photomicrographs (Figures 5C,H,M). These cell pictures were then converted into binary images (Figures 5D,I,N), allowing for skeleton and fractal analyses. Representative skeleton test images (Figures 5E,J,O) display branches in red, junctions in blue and endpoints in green. Then, quantification of the morphological changes was performed, and the number of branches, junctions and end points were assessed using a skeleton analysis (Figure 5P). This analysis showed a significant reduction in the number of branches in the ONC group (*p* < 0.001) in comparison to the control. Similarly, the ONC + GH group exhibited a significant decrease (*p* < 0.01) against the sham group, although GH treatment significantly increased (*p* < 0.05) the number of branches compared to the ONC group. In regard to junctions, both the ONC (*p* < 0.001) and ONC + GH (0.01) groups showed significant reductions as compared to sham but also showed a significant difference between themselves (*p* < 0.05). In turn, end points analysis also revealed significant reductions in both the ONC and ONC + GH groups (*p* < 0.001) in comparison to the sham control (Figure 5P).

Figure 5Q shows that cell body area was significantly reduced (*p* < 0.001) in both the ONC and ONC + GH groups compared to the sham group. This finding was consistent with the significant decrease (*p* < 0.001) in cell body perimeter of both groups compared to the sham control (Figure 5R). Furthermore, fractal analysis (Figure 5S) demonstrated a significant reduction (*p* < 0.05) in the complexity of the branching pattern (fractal dimension, Db) in the ONC group compared to the sham. Additionally, lacunarity was significantly decreased in both the ONC and ONC + GH groups (*p* < 0.001) compared to the sham. Both ONC and ONC + GH groups also exhibited a significant increase in density (*p* < 0.001) compared to the control. Span ratio analysis showed significant increases in the ONC (*p* < 0.05) and ONC + GH (*p* < 0.01) groups, respectively. However, no significant differences were observed between groups in microglia circularity.

### Microglial morphological changes in the retina 14 days after ONC and GH treatment

**Figure 6:**
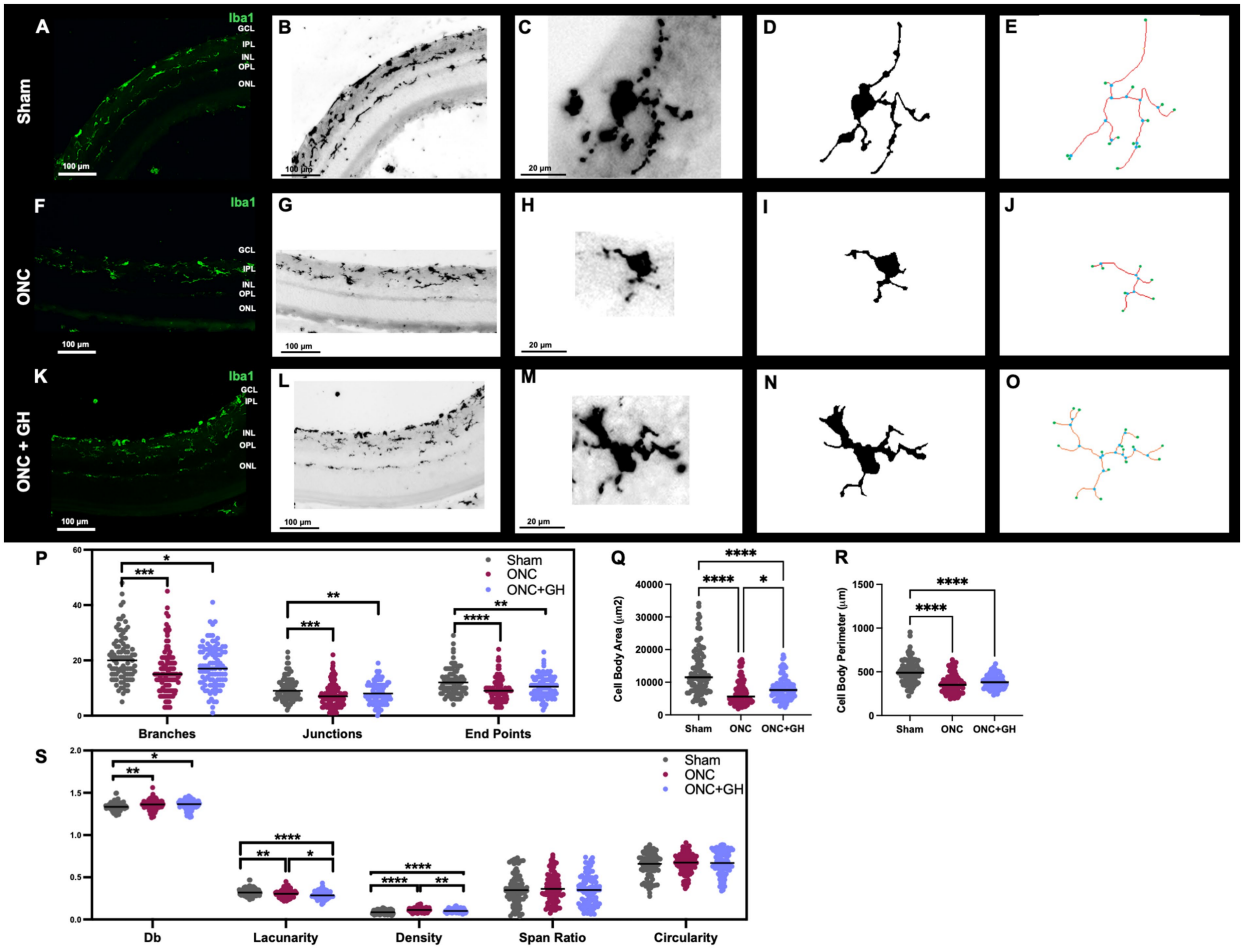
Microglial morphological changes in the rat retina 14 days after ONC and GH treatment, by using Iba1 immunohistochemistry. **(A,B,F,G,K,L)** Iba1 immunoreactivity in the retina; **(C,H,M)** isolated individual microglial cells; **(D,I,N)** binary images of the isolated individual microglial cells; **(E,J,O)** skeletonized images of individual microglia, where branches are in red, junctions in blue and endpoints in green; **(P)** quantification of skeleton analysis, including number of branches, junctions and end points; **(Q)** microglial cell body area; **(R)** microglial cell body perimeter; **(S)** fractal analysis showing fractal dimension (Db), lacunarity, density, span ratio, and circularity of microglia. Studies were performed using skeletal analysis and FracLac plugins in ImageJ. Experimental groups: sham (control), optic nerve crush (ONC), ONC + GH. Results are presented as individual data points with the mean indicated, and each group represented by a different color (*n* = 100 microglia, 6 animals). Asterisks indicate significant differences among groups. ^*^p < 0.05; ^**^p < 0.01; ^****^p < 0.001; ***, ^###^*p* < 0.001, determined by one-way ANOVA followed by Fisher’s LSD as *post-hoc* test.

Morphological analyses were also performed 14 days after ONC (Figure 6) using a similar approach. Iba1 IHC was used to visualize microglial morphology (Figures 6A,B,F,G,K,L). At this time point, Iba1-IR was observed in IPL and OPL in the sham retinas. Again, following ONC, a clear reduction in Iba1-IR was noted in the OPL; however, GH treatment induced an increased immunoreactivity in the OPL. To analyze the morphological changes that occurred with the treatments, individual microglial cells were isolated from the photomicrographs (Figures 6C,H,M) and converted into binary images (Figures 6D,I,N), as described earlier. Figures 5E,J,O show representative skeleton test images, where branches are displayed in red, junctions in blue and endpoints in green. Skeleton analysis (Figure 6P) exhibited a significant reduction in the number of branches in both ONC (*p* < 0.001) and ONC + GH (*p* < 0.05) groups compared to the sham group. Similarly, the number of junctions was significantly decreased in ONC (*p* < 0.001) and ONC + GH (*p* < 0.01) compared to the sham. End points were also significantly reduced in both the ONC (*p* < 0.001) and ONC + GH (*p* < 0.01) group compared to the sham control.

At this time point, cell body area (Figure 6Q) and cell body perimeter (Figure 6R) were significantly reduced in the ONC (*p* < 0.001) and ONC + GH (*p* < 0.001) groups, although GH treatment significantly increased the cell body area compared to the ONC group (*p* < 0.05). Fractal analysis (Figure 6S) demonstrated that ONC significantly reduced (*p* < 0.01) the complexity of the branching pattern (fractal dimension, Db) compared to the sham group, with a similar, though less pronounced, reduction observed in the ONC + GH group (*p* < 0.05). Lacunarity was significantly decreased (*p* < 0.01) by ONC compared to sham, and GH treatment further decreased this parameter compared to both sham (*p* < 0.001) and ONC (*p* < 0.05). Density was significantly reduced in the ONC + GH group (*p* < 0.001) compared to ONC group, while ONC alone caused a significant increase in density compared to both sham (*p* < 0.001) and ONC + GH (*p* < 0.01). Span ratio and circularity did not show significant changes 14 days post-ONC in any of the groups.

### Effects of GH on optic cup diameter at 14 days after ONC and GH treatment

**Figure 7:**
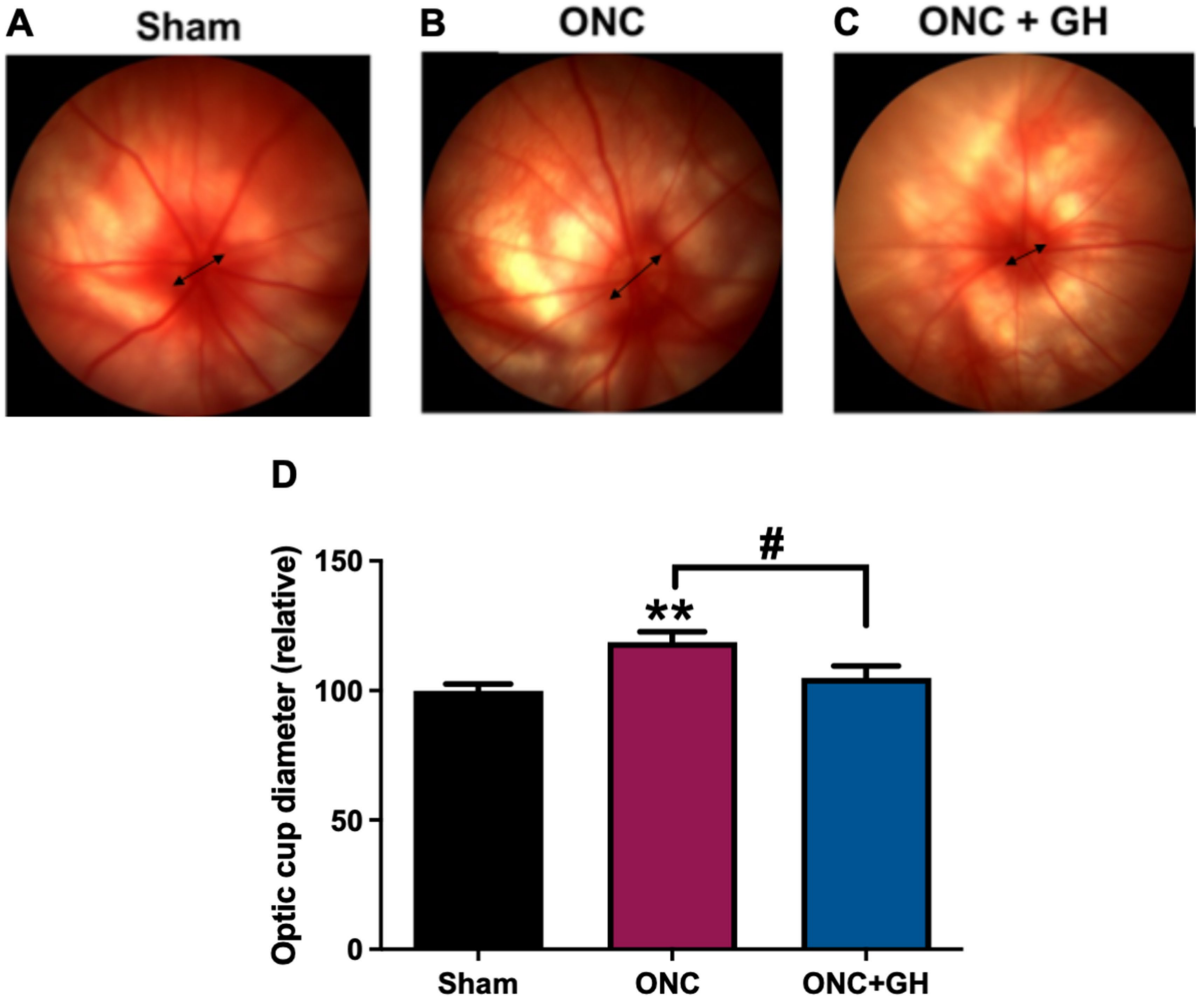
Optic cup diameter changes in the rat retina at 14 days post-ONC and GH treatment. **(A–C)** Fundus photograph of rat at 14 days post-ONC and GH treatment. **(D)** Relative optic cup diameter at 14 days post-ONC and GH treatment. Experimental groups: sham (control), optic nerve crush (ONC), ONC + GH. Results are shown as mean ± SEM (*n* = 8–11); asterisks indicate significant differences compared to control (sham) and number signs indicate multiple comparisons among groups. ^#^*p* < 0.05; ^**^p < 0.01, determined by one-way ANOVA followed by Fisher’s LSD as *post-hoc* test.

Representative fundus images from the Sham, ONC, and ONC + GH groups are shown in Figures 7A–C, respectively. The optic cup diameter was measured and compared between groups (Figure 7D). A significant increase (*p* < 0.01) in the optic cup diameter was observed in the ONC group compared to the sham group. However, GH treatment significantly reduced (*p* < 0.05) the optic cup diameter compared to the ONC group, with no significant difference observed between the ONC + GH group and the sham group.

### Effects of GH on the optomotor reflex 28 days after ONC and 14 days post-GH treatment

**Figure 8:**
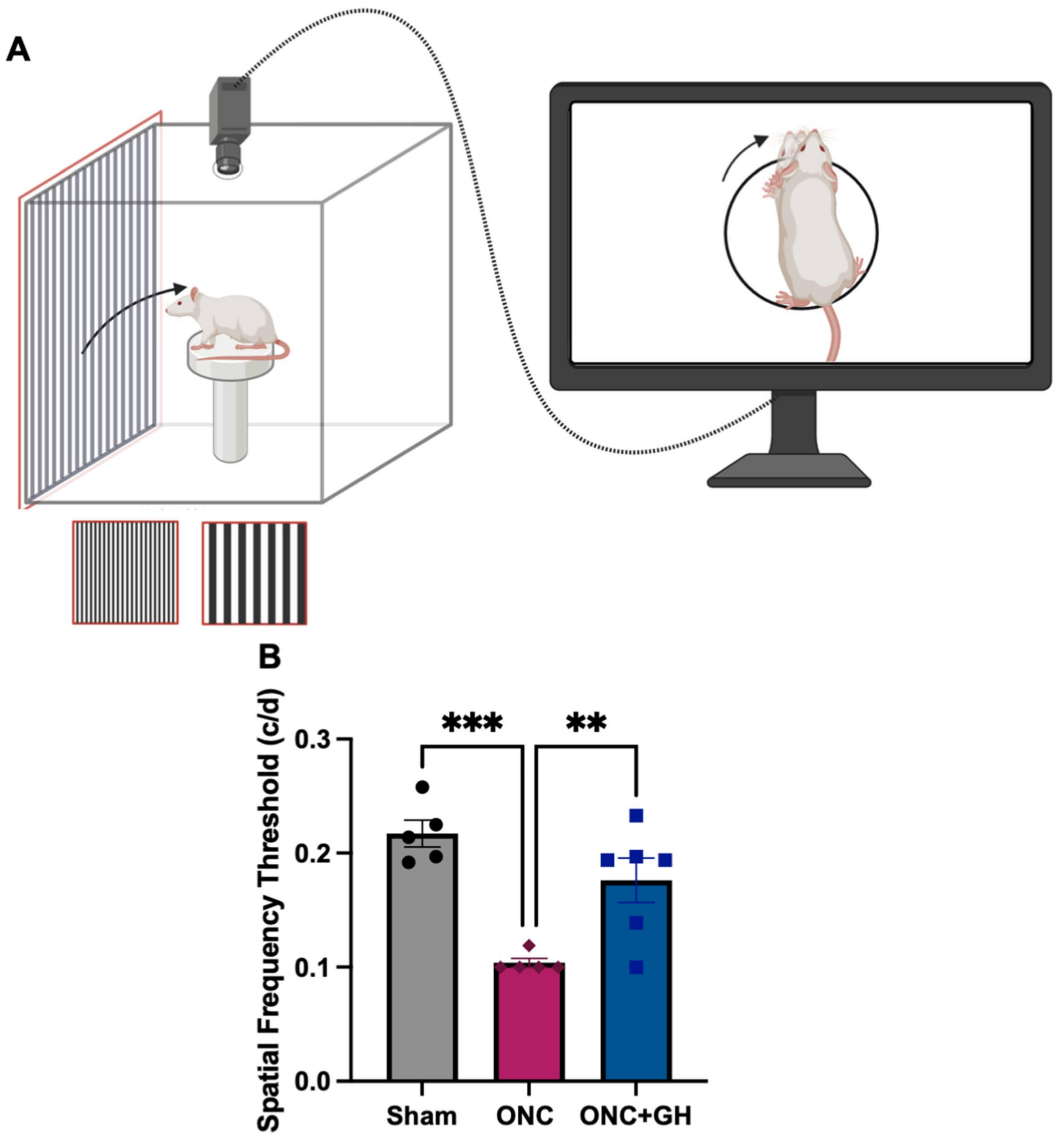
Optomotor kinetic tracking (OKT) changes at 28 days after ONC, and 14 days after GH treatment. **(A)** Optomotor reflex test methodology; **(B)** OKT response at 28 days after ONC. Data are expressed as mean ± SEM (*n* = 5–6). Asterisks indicate significant differences by multiple comparisons among groups. ^**^p < 0.01; ^***^p < 0.001, determined by one-way ANOVA followed by Fisher’s LSD as *post-hoc* test. Created in BioRender.com. (Balderas, 2025).

As a measure to determine the effect of treatments on visual function, the optomotor kinetic tracking (OKT) response to a visual stimulus was analyzed using the OptoMotry system (Figure 8A). The ONC group exhibited a significant decrease of the spatial frequency threshold compared to the sham group (*p* < 0.001) (Figure 8B). In fact, the ONC lesioned rats did not respond to the visual stimuli, and their readings remained at baseline throughout the test. In contrast, the lesioned GH-treated group showed no significant differences from the sham group but exhibited a significant increase in the spatial frequency threshold compared to the ONC group (*p* < 0.01).

### Effect of GH treatment on cytokine expression and NF-κB signaling

**Figure 9:**
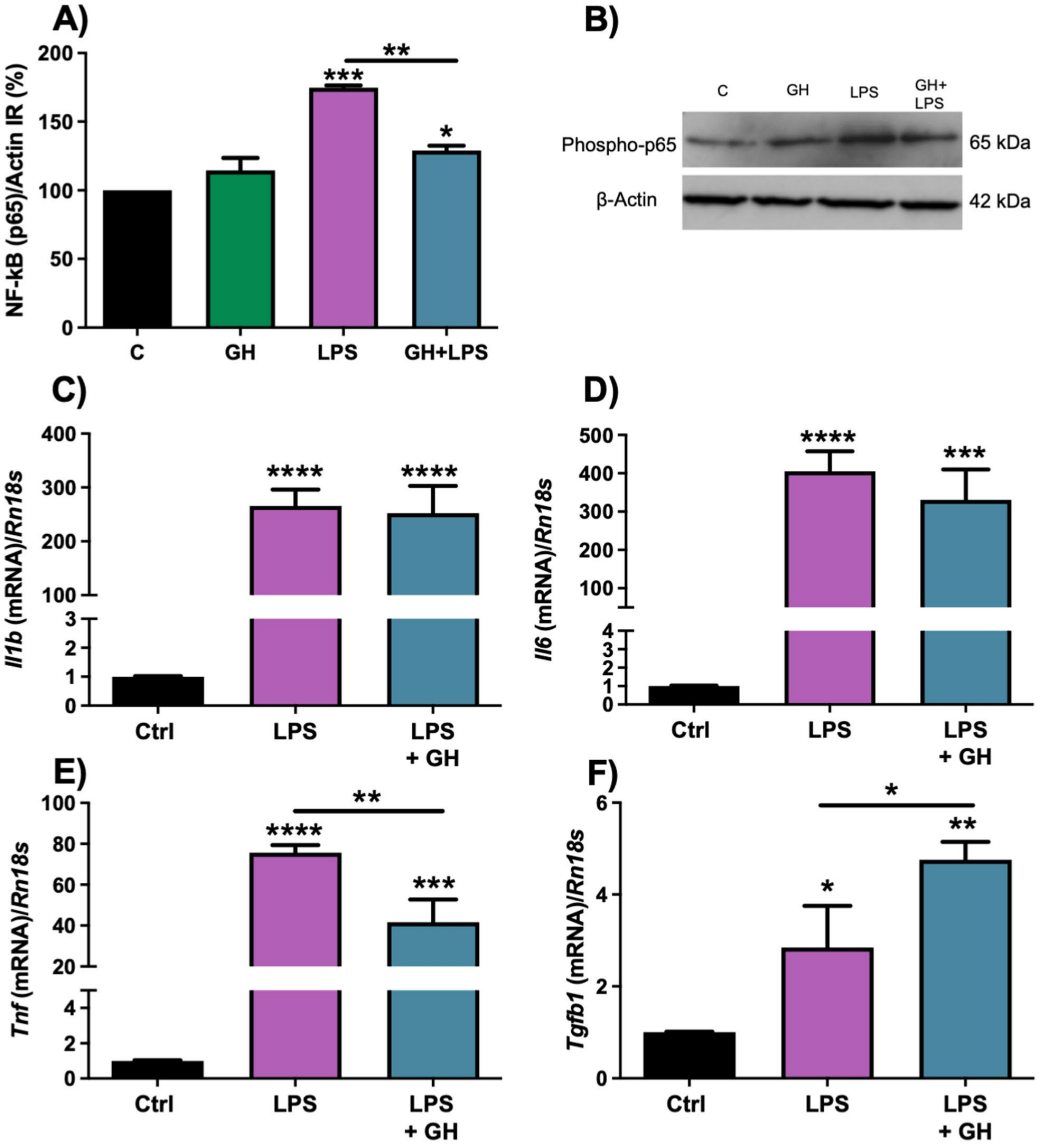
Determination of NF-kB signaling by WB, and cytokines mRNA expression by qPCR, in cultures of SIM-A9 microglial cell line after 6 h of LPS-induced inflammation and GH treatment. **(A)** Densitometric analysis of phospho-p65/β Actin ratio. **(B)** Representative Western blot luminograms of phospho-p65 and β Actin immunoreactivity in SIM-A9 cells 6 h after LPS stimulation and GH treatment. β Actin re-blotting was used as loading control. **(C–F)** Relative expression levels of *Il1b, Il6, Tnf*, *and Tgfb1* mRNAs, respectively, at 6 h post-treatment. Experimental groups: control (ctrl), LPS, LPS + GH. 18 s was used as the reference gene. Results are presented as mean ± SEM (*n* = 4); asterisks indicate significant differences compared to the control and number signs indicate multiple comparisons among groups. ^*^p < 0.05; ^**^p < 0.01, ^***^p < 0.001, ^****^p < 0.0001, determined by one-way ANOVA followed by Fisher’s LSD as *post-hoc* test.

To complement our study, we aimed to analyze the anti-inflammatory effect of GH in response to an LPS challenge using a mouse microglia-derived SIM-A9 cell line. Figures 9A,B show that LPS-treatment significantly activated (*p* < 0.001) the NF-κB signaling pathway, as evidenced by increased p65 immunoreactivity determined by Western blot, in comparison to the sham group; whereas GH treatment significantly reduced (*p* < 0.01) p65 phosphorylation levels as compared to the LPS group, although was still different (*p* < 0.05) than the sham control. Additionally, cytokine expression analysis by PCR showed that LPS stimulation significantly upregulated *Il1β* (*p* < 0.001) (Figure 9C), *Il6* (*p* < 0.001) (Figure 9D), *Tnf* (*p* < 0.001) (Figure 9E), and *Tgfb1* (*p* < 0.05) (Figure 9F) mRNAs. GH treatment significantly reduced *Il6* expression, lowering its significance from *p* < 0.0001 in the LPS group to *p* < 0.001 in the LPS + GH group in relation to sham group; the direct comparison between these groups showed no significant difference (*p* = 0.57). Regarding *Tnf* expression, a significant reduction was observed following GH treatment (*p* < 0.01) compared to the LPS group. Conversely, GH further increased the expression of the anti-inflammatory cytokine *Tgfb1* (*p* < 0.05). It is important to note that GH treatment only slightly increased the difference between the LPS and LPS + GH groups; however, both groups remained significantly different from the sham group.

## Discussion

This study shows that the neurotrophic effects of GH include a significant modulatory role in local retinal inflammation and microglial response, potentially enabling neuroprotection after ONC-induced damage. Moreover, it was observed that GH treatment preserved the optic cup diameter and improved the optomotor reflex in this experimental lesion model. In the CNS, GH is known to exert neuroprotective and neuroregenerative effects, including increased cellular viability and reductions in apoptosis and necrosis (Alba-Betancourt et al., 2013; Bianchi et al., 2017). GH also exhibits antiinflammatory effects, such as reducing inflammatory mediators, promoting neurotrophic factor expression, and controlling disease progression to facilitate resolution of inflammation (Baltazar-Lara et al., 2022; Huang et al., 2022). In the retina, GH has been associated with neuroprotective and neuroregenerative effects following excitotoxic or ONC-induced damage, where it promotes the release of growth factors, an increase in synaptogenic factors expression and improves the ERG pattern (Fleming et al., 2018; Martinez-Moreno et al., 2018; Epardo et al., 2024).

Retinal microglia play a critical role in neural plasticity, neurogenesis, synaptic pruning, and the maintenance of retinal structure and function, since they actively modulate inflammatory responses, phagocytosis and retinal angiogenesis (Checchin et al., 2006; Wang et al., 2016; Rathnasamy et al., 2019; Fan et al., 2022). In response to inflammatory conditions, microglia assume an activated state characterized by morphological changes and the acquisition of either a classical proinflammatory phenotype (M1) or an alternative antiinflammatory phenotype (M2), leading to altered expression of activation markers such as *Aif1* and phenotype specific markers (M1: CD86; M2: CD206) (Jha et al., 2016; Zhou et al., 2017; Guo et al., 2022). Our findings suggest that GH mitigates microglial activation by modulating both phenotypic expression (proinflammatory M1 and antiinflammatory M2) and cellular morphology, a combination that is critical in the retinal inflammatory response. At 24 h post-lesion, qPCR analysis showed increased expression of specific microglial markers (*Aif1*, *Cd86*, and *Mrc1*) in the ONC group, whereas GH treatment showed a tendency to decrease the expression of these indicators as shown in Figure 2. These effects are similar to those observed with certain anti-inflammatory drugs used to treat retinal diseases (Zhou et al., 2007a; Zhou et al., 2007b; Zhang et al., 2018). However, these gene expression changes were not mirrored at the protein level when assessed by WB at this temporal stage (Figures 4D–F). This discrepancy may indicate specific regulation at the translational level or rapid protein degradation. Nonetheless, the observed trend aligns with the changes in gene expression.

Furthermore, a reduction in gene-expression of microglial markers may be associated with a parallel decrease in proinflammatory cytokines. In support of this, we observed that GH treatment following ONC significantly reduced the expression of *Il6*, *Tnf*, *Il18*, and *Nos2* at 24 h (Figure 2A), which was corroborated by ELISA data showing a decrease in TNFα and a similar pattern for *Il6* (Figures 4A,B). These findings correlate with previous studies associating reduced CNS inflammation with improved neurological function and protection against neural damage (Lloyd et al., 2008; Becher et al., 2017). Importantly, these reductions persisted up to 14 days post injury, indicating a sustained effect of GH on proinflammatory cytokines (Figure 2B). While long-term cytokine suppression could, in some contexts, increase vulnerability to neural damage and impair neurotrophic system function (Muñoz-Fernández and Fresno, 1998; Song and Wang, 2011), our results suggest that GH treatment maintains a protective balance, enhancing neurotrophic function through neuroimmune modulation (Epardo et al., 2024).

We also observed that TNFα receptors expression (Figure 2A), which increased at 24 h after ONC as part of the expected acute inflammatory response, was attenuated by GH treatment. This reduction in TNFα activity aligns with reports relating lower TNFα levels with neuroprotective outcomes in neuroinflammation models (Probert et al., 2000; Balzano et al., 2020; Papazian et al., 2021). Importantly, this modulation of TNFα receptor expression appeared to be transient, as no significant changes were observed by 14 days post-ONC (Figure 2B).

Regarding angiogenesis and related factors, we observed an increase in *Ptgs2* expression at 24 h post injury (Figure 2A), consistent with acute inflammatory state and angiogenesis linked to oxidative stress (Lee et al., 2006; Font-Nieves et al., 2012). Although GH has been associated with angiogenic effects (Gould et al., 1995; Struman et al., 1999), we observed that GH induced a tendency to reduce *Vegfa* at 24 h (Figure 2A) and a significant decrease at 14 days (Figure 2B). This indicates a possible sustained antiangiogenic effect, which could be dependent on the molecular form and specific context of GH administration as previously described (Corbacho et al., 2002; Shaker et al., 2023).

The morphological analysis of microglia further supports GH antiinflammatory effects in the retina after an ONC. Following injury, microglia exhibited noticeable morphological changes indicative of a proinflammatory state, transitioning from highly ramified cells to ameboid forms with reduced branching complexity (Soltys et al., 2001; Morrison and Filosa, 2013; Morrison et al., 2017; Gullotta et al., 2023). We observed that ONC led to a significant reduction in the number of microglial branches, junctions and end points, typical markers of an inflammatory response (Illes et al., 2020; Gullotta et al., 2023). Interestingly, GH administration counteracted these changes by preserving the number of branches and junctions at 24 h post-ONC (Figure 5P), indicating that GH treatment promotes a more stable, less inflammatory microglial morphology. These protective effects parallels findings observed with other antiinflammatory agents such as Runx1 and Iptakalim, which similarly prevent extensive microglial activation (Zhou et al., 2007a; Zusso et al., 2012). However, by 14 days post-ONC, both the ONC and GH groups exhibited decreased branching, junctions and end points, with more pronounced reductions in the ONC groups. While microglia in the GH group also showed a reduction, it was notably less severe compared to the injured group (Figure 6P). This sustained, yet moderate, effect with GH treatment is consistent with previous studies showing that specific antiinflammatory agents or morphology inhibitors reduce microglial activation (Nutile-McMenemy et al., 2007; Hinwood et al., 2013).

Analysis of cell body area and perimeter provided additional insights into the modulation of microglial response of GH treatment. Following ONC, microglial cells displayed a decrease in cell body area and perimeter, associated with reduced branching and smaller soma characteristic of an inflammatory response (Karperien and Jelinek, 2015; Green et al., 2022). While GH treatments did not significantly alter these parameters at 24 h post-ONC (Figures 5Q,R); however, by 14 days, GH partially reversed the ONC induced decrease in the cell body area, suggesting some level of recovery from the damage (Figure 6Q). Despite unchanged perimeter, this increased area may reflect the initiation of a reparative response that promotes morphological restoration. Fractal analysis, which quantifies cell complexity based on morphological patterns, also demonstrated a protective GH effect. In the ONC group, fractal dimension (Db), correlating with branching complexity, significantly decreased, indicating a morphological simplification typical of reactive microglia (Karperien and Jelinek, 2015; Morrison et al., 2017; Green et al., 2022). However, GH treatment preserved fractal dimension levels close to those in the sham group at 24 h post-ONC (Figure 5S). This suggests that GH supports the maintenance of a more complex, less reactive microglial structure post-ONC, counteracting the morphological simplification that accompanies inflammation (Karperien et al., 2013; Karperien and Jelinek, 2015). At 14 days post-ONC, GH treatment continued to modulate microglial morphology by a decrease in fractal dimension compared to the ONC group, potentially indicating a hyper-ramification by ONC group as a response to the chronic stress which in some cases ends up in an increment of fractality (Hinwood et al., 2013; Karperien et al., 2013). Lacunarity, a geometric measure of how pattern fills space and thus its visual texture, reflects the degree of heterogeneity of the cell (Soltys et al., 2001; Karperien and Jelinek, 2015). In our ONC model, decreased lacunarity was observed at 24 h with no significant changes by the GH treatments at this early time point. This aligns with prior research indicating that microglial activation under inflammatory conditions often results in reduced lacunarity (Karperien and Jelinek, 2015). At 14 days post-ONC, lacunarity remained lower in the ONC group, while GH treatment induced a further decrease relative to the ONC group. This reduction in lacunarity may correlate with the elevated fractal dimension observed in the ONC group, suggesting an ongoing structural complexity in the microglial pattern that GH treatment may be modulating with a less reactive morphology.

Additionally, pixel density, a measure by the number of pixels per unit area, and span ratio, the proportion of the longest length to the longest width, were analyzed. Both metrics increased in the ONC group at 24 h without significant alteration by GH treatment. Increased density suggests more compact phenotype, which may be related to the decreased area and perimeter. Also, increased span ratio, suggested that microglia were more de-ramified which means more rod like shape, this phenotype have been described as highly proliferative (Vega-Torres et al., 2022). At 14 days, density remained elevated by ONC, whereas GH treatment significantly reduced it, indicating a possible return to a less activated state in microglia acquiring a less compact phenotype (Figure 6). Conversely, the initial changes in the span ratio were no longer significant at 14 days, suggesting a temporary effect of ONC on cell elongation, and that proliferative phenotype is necessary in acute inflammatory state. The circularity of microglial cells, a parameter reflecting cell shape, showed no significant changes across conditions or time points (Figures 5, 6).

The observed effects of GH on retinal inflammation and microglial morphology indicate a potential neuroprotective role following ONC. GH treatment downregulates proinflammatory responses and preserves a less activated microglial morphology, as shown by the maintenance of microglial branches and endpoints shortly after ONC. At 14 days post-ONC, GH continues to moderate microglial reactivity sustaining more balanced morphology. These finding suggest that GH modulates both early and sustained inflammatory responses, possibly preserving retinal structure and function post-ONC. In line with this, the inhibition of proinflammatory cytokine release by GH appears to be related to the modulation of microglial activation, phenotype markers, and overall morphology. Microglial phagocytosis is increasingly recognized as a critical mechanism in maintaining retinal homeostasis and shaping the balance between inflammatory and regenerative responses. In this context, GH-mediated modulation of microglial activity may extend beyond cytokine regulation to also influence phagocytic activity. Although this was not directly assessed, the conceptual integration of phagocytic capacity into the neuroprotective role of GH highlights a promising avenue for future research.

Given that the retina is a complex tissue where glial cells, various neuronal types, and components of the microvasculature interact in a coordinated manner and considering that inflammatory response regulators can also be produced by other glial cells such as Müller cells and astrocytes, we aimed to correlate our *in vivo* morphological findings with the effects of GH on SIM-A9 microglial cells. These cells were activated with LPS to induce a pro-inflammatory phenotype. We observed a significant reduction in the phosphorylation of the p65 subunit, indicating that activation of the GH receptor (GHR) attenuates NFκB signaling and can counteract the LPS-induced expression of TNFα and IL-6. Furthermore, there was a notable increase in TGFβ levels, a factor associated with a beneficial or neurotrophic microglial phenotype. Previous studies using the SIM-A9 cell line have shown that prolactin (PRL), a hormone structurally and functionally related to GH, exerts similar anti-inflammatory effects to those observed in our study (Jayakumar et al., 2022). The choice of including the SIM-A9 microglial cell line in this study reflects not only practical aspects of culture stability in a model that allows reproducibility and minimizes variability. While cultures of isolated adult rat microglia may provide a better physiological approach, their instability and contamination with fibroblast-like cells pose significant challenges. Thus, the use of SIM-A9 cells offered a consistent way to analyze GH–microglia interactions and it complemented our *in vivo* observations.

Fundoscopy is a non-invasive imaging technique that allows examination of the eye fundus, including the retina, optic disc and blood vessels (Hara et al., 2021). In the case of ONC injury, fundoscopy provides an option to monitor RGCs survival, as changes in optic cup diameter often indicate RGCs loss, providing insights into the extent of damage and potential neurodegeneration (Dervisevic et al., 2013; Hara et al., 2021). The visualization of the optic disc in albino rats lacks the precision observed in clinical ophthalmology, and therefore, the measurement of morphological parameters of the optic nerve head presents inherent limitations. Nonetheless, in our experiments, we were able to detect a clear variation in the apparent size of the optic nerve fiber entry point following compressive injury, as well as a marked positive effect of GH when administered for 14 days post-injury (Figure 7D). In this same model, we recently reported a consistent loss of over 80% of RGCs 2 weeks after ONC, along with a moderate but significant increase in cell survival and a correlation with electroretinogram (ERG) parameters in GH-treated animals (Epardo et al., 2024). We also previously observed an increase in the number of axons labeled with CTB-488 and positive for GAP43, indicating a protective and regenerative effect of GH, which correlates positively with our fundoscopic observations (Epardo et al., 2024). We are aware of the limitations of this technique in murine models; however, it is important to emphasize that the observations were consistent with other quantified indicators and, moreover, this is a non-invasive method that allows for integration with additional structural and functional assessments. This approach has been previously used in fundoscopy studies in rodents (Cohan et al., 2003; Hagen et al., 2022).

The optokinetic response is a compensatory eye movement elicited in the direction of a moving visual stimulus (Thomas et al., 2004). Optic nerve crush (ONC) caused significant long-term deficits, as evidenced by a marked reduction in the optomotor reflex response (Figure 8B). However, GH treatment significantly improved this response compared to the ONC group, reaching levels comparable to those observed in sham-operated animals. It is important to note that the observed recovery of the optokinetic reflex does not necessarily indicate full/partial restoration of vision but rather reflects an enhanced ocular response to photic stimulation. Moreover, GH is known to induce robust neural plasticity at the central level, raising the possibility that the improvement observed in this test may not result from axonal protection or regeneration *per se*. We hypothesize that the residual retinal ganglion cells (RGCs) maintaining connectivity with the superior colliculus or the lateral geniculate nucleus could underlie the partial reflex recovery. Alternatively, the response may be generated from a behavioral adaptation or from compensatory mechanisms involving the contralateral (uninjured) eye. Further experiments are required to elucidate the precise mechanisms underlying this functional improvement, and ongoing studies in our laboratory are currently addressing this question.

Current research is increasingly directed toward identifying and developing therapies that utilize factors with neurotrophic properties in the retina, including the classical neurotrophins BDNF, NT3, and NGF (Kimura et al., 2016; Hill et al., 2021; Abraham and Telias, 2025). Neuroprotective and regenerative effects have also been reported for various growth factors, steroids, and vitamins. Among the growth factors with well-documented potent effects on retinal and optic nerve regeneration and protection are CNTF and GDNF (Kimura et al., 2016). In addition, certain hormones, such as GnRH, PRL, TRH, and GH, have also shown promising effects on retinal regeneration (Díaz-Galindo et al., 2020). Some signaling pathways, such as JAK/STAT, PI3K/Akt, and MAPK, are widely recognized for promoting regeneration and protection in the retina; therefore, there are numerous molecular candidates that could potentially activate these intracellular communication routes (Fleming et al., 2019; Lee et al., 2022; Ávila-Mendoza et al., 2023; Ávila-Mendoza et al., 2024).

As mentioned above, this study was performed only in young adult male rats and did not include females. The rationale was that this work follows up a previous study where we demonstrated that GH treatment promoted retinal neuroprotection, stimulated axonal growth and restored electrophysiological function (as determined by electroretinogram), also in male rats injured by ONC (Epardo et al., 2024). We wanted to further investigate the role of GH upon regulation of neuroinflammation, cytokine expression and microglial activity in response to ONC, to better understand its neuroprotective mechanisms, and thus considered it important to keep the same experimental model to diminish potential sources of variation. It is known that estrogens exert potent neuroprotective effects, which could potentially mask the specific actions of GH that we are studying. Moreover, GH treatment can stimulate steroidogenesis in granulosa cells (Ahumada-Solórzano et al., 2016) and alter endogenous estrogen levels. On the other hand, several studies indicate that GH actions are sex-dependent, with males showing stronger responses in the hypothalamic expression of genes such as BDNF and synaptophysin (Dos Santos et al., 2023), while pSTAT5 activation or fear-response behavior exhibit sex-specific patterns (Dos Santos et al., 2023; Menezes et al., 2024). We have previously reported that GH also promotes neuroprotective and regenerative effects in the harmed nervous system of females, as demonstrated in a spinal cord injury (SCI) model; however, these results were obtained in ovariectomized rats, where the effects of ovarian estrogens were eliminated (Martínez-Moreno et al., 2023). Taken together, these findings remark the importance of considering sex differences in GH actions and highlight the necessity for future studies in female models with controlled hormonal status.

In conclusion, our findings demonstrate that GH treatment exerts significant neuroprotective effects in the context of retinal inflammation and ONC induced injury. GH not only modulates microglial response, reducing proinflammatory markers/signaling and preserving a less reactive microglial morphology, but also mitigates changes in density and morphological complexity that typically accompany microglial activation. Additionally, GH appears to play preventive role in RGCs loss, as indicated by the reduction on optic cup diameter and improvements in the optomotor reflex. These results suggest that GH could be a potential therapeutic strategy to mitigate neurodegeneration and promote retinal homeostasis after an optic nerve injury.

## Data Availability

The original contributions presented in the study are included in the article/supplementary material, further inquiries can be directed to the corresponding authors.
